# Plant-Based Biofillers for Polymer Composites: Characterization, Surface Modification, and Application Potential

**DOI:** 10.3390/polym17172286

**Published:** 2025-08-23

**Authors:** Mateusz Pęśko, Anna Masek

**Affiliations:** Faculty of Chemistry, Institute of Polymer and Dye Technology, Lodz University of Technology, Stefanowskiego 16, 90-537 Lodz, Poland; mateusz.pesko@dokt.p.lodz.pl

**Keywords:** fiber surface modification, plant-based biofillers, lignocellulosic reinforcement, sustainable material design, thermoset and thermoplastic matrices, polymer composites

## Abstract

The mounting global concern regarding the accumulation of plastic waste underscores the necessity for the development of innovative solutions, with particular emphasis on the incorporation of plant-based biofillers into polymer composites as a sustainable alternative to conventional materials. This review provides a comprehensive and structured overview of the recent progress (2020–2025) in the integration of plant-based biofillers into both thermoplastic and thermosetting polymer matrices, with a focus on surface modification techniques, physicochemical characterization, and emerging industrial applications. Unlike the prior literature, this work highlights the dual environmental and material benefits of using plant-derived fillers, particularly in the context of waste valorization and circular material design. By clearly identifying a current research gap—the limited scalability and processing efficiency of biofillers—this review proposes a strategy in which plant-derived materials function as key enablers for sustainable composite development. Special attention is given to extraction methods of lignocellulosic fillers from renewable agricultural waste streams and their subsequent functionalization to improve matrix compatibility. Additionally, it delineates the principal approaches for biofiller modification, demonstrating how their properties can be tailored to meet specific needs in biocomposite production. This critical synthesis of the state-of-the-art literature not only reinforces the role of biofillers in reducing dependence on non-renewable fillers but also outlines future directions in scaling up their use, improving durability, and expanding performance capabilities of sustainable composites. Overall, the presented analysis contributes novel insights into the material design, processing strategies, and potential of plant biofillers as central elements in next-generation green composites.

## 1. Introduction

**Figure 1:**
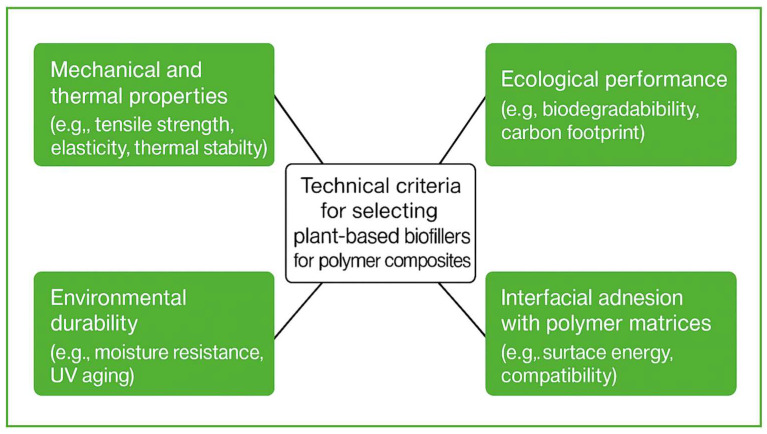
Technical criteria for selecting plant-based biofillers for polymer composites. The diagram outlines key evaluation parameters such as mechanical and thermal properties (e.g., tensile strength, and elasticity), environmental durability (e.g., moisture and UV resistance), interfacial adhesion with polymer matrices (e.g., surface energy compatibility), and ecological performance (e.g., biodegradability and carbon footprint).

In response to the increasing environmental concerns and the urgent need to safeguard natural resources, there is a growing emphasis on developing sustainable alternatives to conventional synthetic materials [1]. This paradigm shift has prompted the exploration of natural materials, particularly those derived from renewable resources, as viable substitutes for petroleum-based plastics in various industrial applications [2]. A particularly encouraging approach involves the incorporation of plant fibers as reinforcements in polymeric biocomposites [3]. These natural fibers offer several environmental and economic advantages over synthetic fibers, such as biodegradability, recyclability, lower density, and improved thermal, electrical, and acoustic insulating properties [4,5,6]. Moreover, the production of plant fibers is less costly and has a lower carbon footprint, which makes them an attractive option in the context of sustainable development. For instance, flax fibers have been utilized since approximately 7000 years ago in ancient Egypt [7]. This extensive history underscores the potential of these fibers not only in traditional applications but also in modern composite materials. Notwithstanding their potential, the widespread utilization of plant fibers in composites is confronted with specific challenges, including diminished durability in comparison to synthetic materials such as carbon or glass fibers, as well as vulnerability to moisture absorption and biological degradation [8,9,10]. Furthermore, plant fibers exhibit significant variability in their chemical composition and physical properties due to factors such as cultivation conditions, harvesting time, and post-processing methods. This heterogeneity presents a major challenge in ensuring consistent performance of plant-fiber-reinforced composites and limits their scalability for industrial applications. An additional promising trajectory in the development of sustainable materials is the integration of bioplastics. Bioplastics derived from renewable plant- or animal-based sources, such as cellulose, lignin, chitosan, and biopolymers like PLA (polylactic acid) and PHA (polyhydroxyalkanoates), offer the advantages of biodegradability and a reduction in carbon emissions. These biocomposites have the potential to make a substantial contribution to the mitigation of environmental issues associated with conventional plastics. Moreover, the incorporation of plant fibers as reinforcements in these materials enhances their mechanical properties and sustainability profile, rendering them suitable for a variety of applications, ranging from the automotive and packaging industries to biotechnological fields [4,8,11]. Despite the growing interest in biocomposites, the current literature often lacks comprehensive comparative analysis of fiber surface modification techniques, fiber–matrix interfacial behavior, and compatibility with various polymer matrices, including thermoplastics and thermosets. Addressing this gap is essential to advancing the performance and applicability of plant-fiber-based composites. This review focuses on the role of plant fibers as biofillers in polymeric biocomposites. A comprehensive understanding of the chemical structure and physical properties of these materials is paramount for effective selection. As illustrated in Figure 1, the selection of biofillers for biocomposites is influenced by several key factors, including physical properties, durability, and environmental impact. This diagram underscores pivotal parameters such as density, strength, biodegradability, and performance in various operational conditions [6,12].

**Figure 2:**
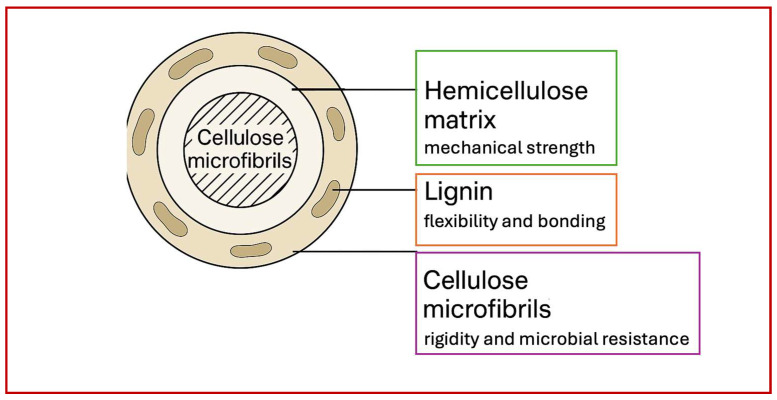
Schematic representation of the supramolecular organization of plant fibers, illustrating the spatial distribution and functional roles of key biopolymers. Highly crystalline cellulose microfibrils are embedded in a matrix of amorphous hemicellulose, which facilitates flexibility and polymer interaction. Lignin is distributed within the matrix, causing rigidity, resistance, and microbial degradation by forming a cross-linked, hydrophobic network.

Another important aspect is the chemical structure of plant fibers, including their main components, such as cellulose, hemicellulose, and lignin. Figure 2 presents these fundamental structural elements of plant fibers. The diagram shows how these three primary components—cellulose, hemicellulose, and lignin—work together to form the fiber structure. Cellulose is responsible for the strength of the fibers, hemicellulose contributes to their flexibility, and lignin provides additional stability and resistance to biodegradation. These interactions create a material with unique properties, making it suitable for various types of biocomposites [13].

**Figure 3:**
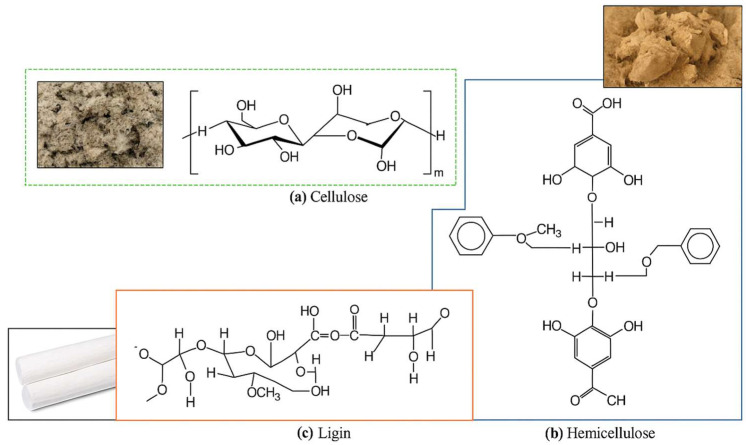
Chemical structures and functional roles of major constituents in plant fibers: (**a**) cellulose linear polysaccharide providing high tensile strength due to β-1,4-glycosidic linkages; (**b**) hemicellulose a branched heteropolymer that enhances flexibility and facilitates matrix adhesion; and (**c**) lignin a phenolic macromolecule responsible for structural rigidity, UV stability, and microbial resistance. These components collectively determine the physicochemical behavior of lignocellulosic biofillers in polymer composites.

Figure 3 presents the chemical structures and functional roles of the three principal components of plant fibers—cellulose, hemicellulose, and lignin—highlighting their significance in composite reinforcement. Cellulose, a linear polysaccharide composed of β-1,4-linked D-glucose units, forms highly crystalline microfibrils that are primarily responsible for the high tensile strength and stiffness of natural fibers. Hemicellulose, in contrast, is an amorphous and branched heteropolymer that contributes to fiber flexibility and enhances compatibility with polymer matrices through increased surface reactivity. Lignin is a complex, cross-linked aromatic macromolecule that reinforces the fiber structure, confers resistance to microbial degradation, and provides thermal and UV stability. The synergistic interaction of these components dictates the mechanical behavior, durability, and interfacial properties of biofillers in polymer composites, making their understanding essential for the rational design of high-performance biocomposites [14,15,16].

This review aims to fill this gap in the literature by providing a critical synthesis of current research on plant-based biofillers, with particular emphasis on chemical structure, surface modification, processing compatibility, and their influence on composite properties. The novelty of this work lies in its integration of physical, chemical, and application-oriented perspectives, which has not been systematically addressed in prior reviews. By addressing the relationships between fiber structure, modification methods, and resulting composite performance, this article contributes to the rational design of biofiller–polymer systems for future material innovation [17]. The aim of this review is to highlight the role of plant-based biofillers in polymeric biocomposites, focusing on their structural and chemical properties and their potential applications across various industries. Plant fibers, with their unique properties, hold significant potential to become a key element in the development of sustainable materials that not only meet technical requirements but also contribute to the protection of the natural environment [18].

## 2. Plant Fiber Integration in Polymer Composites: Applications, Matrix Compatibility, and Mechanical Performance

### 2.1. Applications of Plant Fibers in Polymer Composites and Waste-Derived Fiber Sources

In response to the global challenges associated with environmental degradation and the necessity for sustainable development, the search for alternatives to traditional synthetic materials has become a crucial issue in contemporary materials research [19]. In particular, plant-based fiber-reinforced composites present an ecological alternative to synthetic fibers, gaining increasing significance across various industrial sectors. Plant fibers such as flax, hemp, and ramie are particularly recognized for their relatively high specific tensile strength and stiffness, which, when combined with low density and biodegradability, make them suitable reinforcements in lightweight composite materials [20]. The application of plant-based fibers in polymer composites has gained wide recognition over the past few decades. These composites offer improved sustainability, a reduced carbon footprint, and lower cost compared to synthetic-fiber-reinforced polymers, while also offering functional properties such as biodegradability and biocompatibility. Among the most commonly used plant fibers in composite production are bast fibers, such as jute, hemp, kenaf, flax, and sisal [21,22]. Due to their high cellulose content and low density, these fibers demonstrate a high specific strength-to-weight ratio [23]. Certain plant fibers, such as flax, hemp, and kenaf, exhibit a high specific tensile strength and favorable modulus of elasticity, which when combined with their low density and biodegradability render them competitive with synthetic reinforcements in selected applications. The key mechanical properties, density, and cellulose content of these fibers are summarized in Table 1 [24]. This table presents a comparative analysis of the tensile strength, modulus of elasticity, density, and cellulose content of these fibers, which are crucial for determining their suitability in biocomposite applications [25].

**Figure 4:**
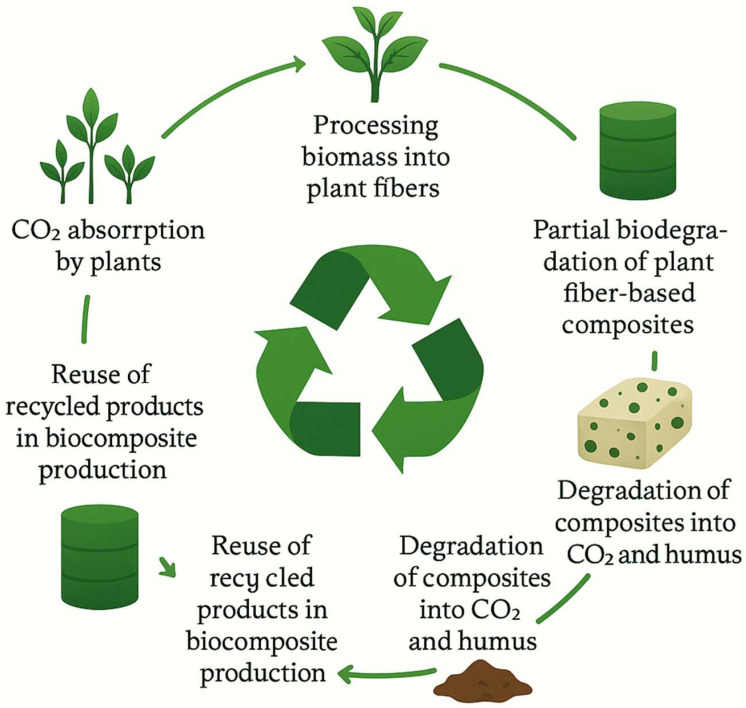
Life cycle model of plant-based fiber-reinforced polymer composites, highlighting carbon sequestration, fiber sourcing, manufacturing, the use phase, biodegradation, recycling, and reintegration into production.

Due to their availability and exceptional mechanical properties, plant fibers present an attractive option for producing high-performance materials that can be applied across various industries. However, despite their promising potential, the use of plant fibers in composites still faces challenges related to their industrial scalability. Most of these materials remain at the laboratory stage due to limited efficiency and technological compatibility with industrial processing [27]. Challenges include high moisture absorption, inconsistent fiber morphology, and poor adhesion to hydrophobic matrices, which hinder processing repeatability and structural reliability. Therefore, only a limited number of plant fibers have been successfully processed at the industrial level, particularly in high-demand sectors such as the automotive industry, where fibers sourced from agronomic processes or post-consumer reuse have already been integrated into final products. Understanding the chemical composition of plant fibers is essential for their effective utilization in biocomposites. The literature identifies key components of these fibers, such as cellulose, hemicellulose, and lignin, which play a fundamental role in their mechanical properties and biodegradability. Cellulose provides structural stiffness and tensile strength, hemicellulose contributes to bonding and flexibility, while lignin enhances resistance to microbial attack and UV degradation. These components provide the basis for the development of new methods for processing plant fibers and integrating them into polymer matrices, leading to the creation of new, efficient, and sustainable materials [28,29,30,31]. Research on cellulose fibers focuses on analyzing their chemical, mechanical, dielectric, and thermal properties using advanced techniques such as X-ray diffraction (XRD), thermal gravimetric analysis (TGA), scanning electron microscopy (SEM), differential scanning calorimetry (DSC), and dynamic mechanical analysis (DMA). These techniques provide precise insights into the properties of the fibers, which is critical for the development of optimal processing methods and practical applications. An important area of research is also the optimization of the interface properties between plant fibers and the polymer matrix in composite materials [32,33]. One of the main challenges is improving the bonding strength between the fiber and the polymer matrix, which directly impacts the overall mechanical performance of the composite. Consequently, much research focuses on modifying fiber surfaces through thermal and chemical treatments, which aim to improve adhesion between the fiber and matrix, as well as reduce water absorption by plant fibers [34]. Alkaline treatments remove surface impurities and increase surface roughness, silane coupling agents improve chemical bonding, and acetylation reduces hydrophilicity, all enhancing interfacial load transfer. The sustainable development of plant-fiber-based composites goes beyond their mechanical properties. An essential aspect of this industry’s growth is the effective management of agricultural waste and the use of recycled materials. In recent years, there has been an increasing interest in utilizing agricultural residues (such as rice husks, straw, and plant stems) for biocomposite production, which plays a crucial role in the circular economy. Utilizing these residues reduces dependence on virgin biomass, lowers production costs, and minimizes landfill pressure, while aligning with zero-waste manufacturing principles. The incorporation of such feedstocks reduces environmental burdens and mitigates material waste, while also addressing the issue of surplus agricultural residues [35,36,37]. The properties of natural fiber-reinforced polymer composites encompass a wide range of matrices, including thermoplastics (polypropylene (PP), polyethylene (PE), polystyrene (PS), and polyvinyl chloride (PVC), thermosets (polyester, epoxy, etc.), rubbers (styrene-butadiene rubber, natural rubber, etc.), and composites based on cement and gypsum [38]. The ability to combine these fibers with various matrices enables the creation of composites with tailored mechanical, thermal, and environmental properties, making them suitable for a wide range of industrial applications. Additionally, processing technologies and composite parameters, such as fiber length, orientation, volume fraction, and surface modifications, significantly influence the mechanical properties of biocomposites. One of the major advantages of using plant-fiber-based composites is their reduced carbon footprint and minimal environmental impact during production and disposal, as shown in Figure 4.

These materials not only replace petroleum-based reinforcements but also contribute to life cycle emissions reduction and improved sustainability metrics across product chains. These benefits, combined with improved mechanical properties, make plant-fiber-based biocomposites a competitive alternative to traditional synthetic materials [39,40,41,42].

The use of theoretical models to predict the properties of these materials is an important tool for the further development of biocomposites. Micromechanical models, such as those presented by Berk and colleagues, account for statistical variations inherent in plant fiber reinforcements, enabling more accurate predictions of the tensile strength and modulus of composites [43]. Such modeling supports the rational design of composites by optimizing fiber–matrix ratios, predicting mechanical performance, and guiding experimental validation of industrial formulations. These studies provide valuable information that can be used to refine biocomposite production processes and optimize their mechanical and environmental properties [44].

### 2.2. Matrix Selection and Compatibility in Biocomposites

Biocomposites are manufactured by combining plant-based fibers with polymer matrices to form sustainable alternatives to synthetic composites [45]. Plant fibers such as flax, jute, hemp, and sisal are chosen for their high specific strength, low density, renewability, and biodegradability [2,37,46]. The polymer matrix plays a crucial role in determining the composite’s mechanical properties and processing behavior. Matrices are broadly categorized into thermoplastics and thermosets, each with distinct thermal, chemical, and recyclability profiles [38,39,40,47]. Thermosetting matrices (e.g., epoxy, unsaturated polyesters, phenolic resins) [48] exhibit high dimensional stability, chemical resistance, and strong fiber–matrix bonding [49,50]. However, they are typically brittle and non-recyclable, making them less favorable for applications requiring circular life cycles [51]. Thermoplastic matrices (e.g., PP, PE, PLA, PHA) offer advantages such as reprocessability, weldability, and ease of shaping post-processing [52,53]. They are ideal for applications demanding flexibility and recyclability but generally require higher processing temperatures and offer lower thermal resistance. Table 2 summarizes the critical differences between these matrix classes in the context of biocomposite performance [54]. 

### 2.3. Mechanical Performance of Plant-Fiber-Reinforced Composites

Plant fibers possess a favorable strength-to-weight ratio and serve as sustainable reinforcements in polymer matrices. Their mechanical performance is primarily influenced by the structural properties of cellulose microfibrils and the presence of hemicellulose and lignin. Cellulose contributes to stiffness and tensile strength through crystalline alignment, while hemicellulose enables interfacial interaction and flexibility. Lignin, being hydrophobic and aromatic, provides dimensional stability and biological resistance. The final composite properties depend on parameters such as fiber loading, aspect ratio, orientation, and the fiber–matrix interface. Processing methods and surface modification techniques (alkaline, silane, acetylation) play essential roles in reducing hydrophilicity and improving adhesion. As shown in Table 2, thermosets typically offer better fiber bonding and thermal performance, while thermoplastics provide benefits in recycling and post-use processing. In summary, effective fiber–matrix selection, interfacial engineering, and structure optimization are critical to the mechanical reliability and sustainability of plant-fiber-reinforced composites.

## 3. Advanced Processing Techniques for Plant-Fiber-Reinforced Composites

The manufacturing of plant-fiber-reinforced composites requires careful adaptation of traditional composite processing techniques to accommodate the unique hygroscopic, heterogeneous, and anisotropic nature of lignocellulosic fibers [56]. Depending on the fiber form such as fabrics, mats, or chopped fibers and their orientation (random or aligned), different processing routes can be employed to optimize material performance [57]. Fiber alignment, aspect ratio, surface treatment, and moisture content are all critical factors influencing final composite properties, including strength, dimensional stability, and void content [58]. In thermoset-based composites, manual methods such as hand lay-up are often used due to their simplicity and adaptability for small-scale production [59]. This method, however, poses challenges related to air entrapment, resin distribution, and reproducibility, as it is highly operator-dependent [41,42]. To address these issues, vacuum-assisted resin transfer molding (VARTM) has been introduced. In this process, resin is drawn into the fiber mats under vacuum pressure, which significantly reduces air contamination and allows for more precise control over fiber spacing and composite geometry [43]. VARTM also supports the fabrication of large-scale, high-performance parts by minimizing fiber misalignment and enhancing surface finish. A study conducted by Franzucci et al. [60] revealed that the swelling and fluid absorption characteristics of natural fibers reduce permeability in saturated conditions. Increased resistance to flow and reduced porosity hinder resin infusion, especially under high-fiber-volume configurations. Masoodi et al. [60] demonstrated, using finite element (FE) modelling, that LCM-based flow simulations can accurately predict resin behavior, allowing improved optimization of process parameters and material use. Nguyen et al. [61] further confirmed that LCM methods enable higher fiber volume fractions and lower processing temperatures, improving mechanical performance while reducing the risk of thermal degradation. Additionally, fiber diameter and porosity were shown to be critical factors influencing resin flow, particularly when different resin chemistries are applied. In the processing of thermoplastic matrices, technologies such as injection molding and compression molding are widely used due to their high precision, speed, and suitability for mass production. In injection molding, the fiber–resin compound—usually in pelletized form—is melted and injected into molds under high pressure [62]. Compression molding, on the other hand, involves pressing fiber mats and molten polymer under controlled temperature and pressure to create flat or contoured components. This technique is favored for structural parts in the automotive and construction sectors because of its scalability and low cycle times. Pultrusion is another widely adopted method applicable to both thermoset and thermoplastic systems. It involves continuously pulling fibers through a heated die, where they are impregnated with resin and cured or cooled depending on the matrix type. The result is a high-strength, low-void-content profile with excellent alignment, used in applications such as beams, rods, and utility structures. The main characteristics and typical applications of these processing techniques are summarized in Table 3.

### 3.1. Hand Lay-Up Process in the Production of Fiber-Reinforced Composites

**Figure 5:**
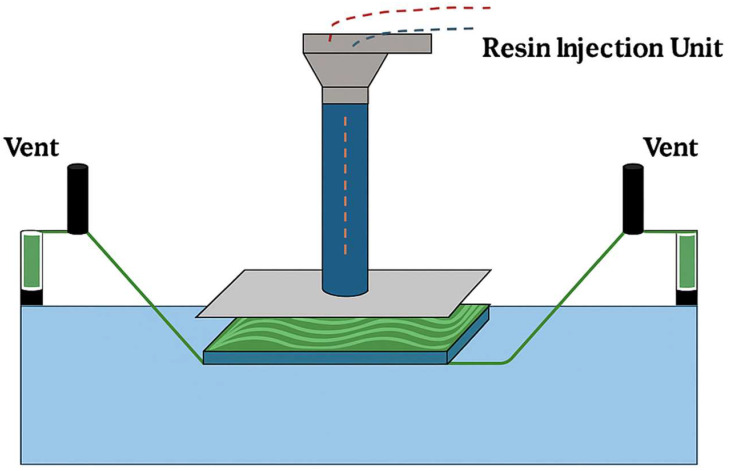
Schematic representation of the hand lay-up process for natural fiber-reinforced composites. The diagram illustrates the key stages: fiber mat placement in an open mold, manual resin application, air removal using rollers, and ambient temperature curing with release film covering.

The hand lay-up process is one of the most traditional and widely used methods for fabricating fiber-reinforced composites, particularly suited for the production of large, custom-shaped parts and prototypes [66,67]. It involves the manual placement of natural fiber fabrics either woven or non-woven into an open mold, followed by the application of a liquid polymer matrix [68]. The resin, typically epoxy or polyester, is applied by brushing or spraying to ensure proper wetting of the reinforcement [69]. Manual rollers are then used to remove trapped air and promote uniform adhesion between fiber layers, minimizing the risk of voids [70]. The composite is typically cured at ambient temperature for up to 24 h, although elevated temperatures may be used to accelerate hardening depending on the resin system [71,72,73]. Commonly used resins in hand lay-up include thermosets such as epoxy, unsaturated polyester, and diphenylmethane diisocyanate (MDI), selected for their compatibility with natural fibers and for imparting appropriate thermal, chemical, and mechanical properties [74]. Despite its manual and labor-intensive nature, the hand lay-up technique remains an economical and effective method for producing large biocomposite components with good mechanical performance and structural integrity [75]. The hand lay-up technique remains one of the most accessible and widely applied methods for fabricating fiber-reinforced composites, particularly in small- and medium-scale production settings. Its effectiveness lies in the simplicity of manually arranging fiber mats, applying the resin, and consolidating the structure with basic tooling, which enables the fabrication of custom geometries and prototyping of biocomposites. However, the method is not without its limitations, such as operator dependency and potential for void formation. To visually support the description of this process and provide greater clarity, a schematic representation of the hand lay-up procedure has been included in this work (Figure 5). The figure illustrates the key stages involved, including fiber placement, resin application, manual consolidation using rollers, and ambient or thermal curing. This visual aid reinforces the operational principles of the method and highlights its relevance in the processing of plant-fiber-reinforced biocomposites.

### 3.2. Vacuum Infusion Techniques in the Production of Plant-Fiber-Reinforced Composites

Vacuum infusion is a widely utilized fabrication method for plant-fiber-reinforced composites and is especially suitable for large parts requiring uniform resin distribution and minimal void content. The process involves the impregnation of dry fiber mats—such as jute, flax, or hemp—with resin under vacuum, which enhances matrix penetration and ensures consistent fiber wetting.

#### 3.2.1. Key Techniques Include

- VARTM (vacuum-assisted resin transfer molding): Resin is drawn into the fiber preform under vacuum. This method is scalable, reduces air voids, and offers high-quality laminate consolidation.
- RIFT (resin infusion under flexible tooling): A general category of resin infusion systems. Type I describes in-plane resin flow (parallel to fibers), and Type II refers to through-thickness flow. Additional types include resin film infusion (Type III) and semipreg infusion (Type XIV).
- SCRIMP (Seemann composite resin infusion molding process): A modified VARTM system that improves resin flow in woven or stitched fabrics by integrating flow-enhancing layers.

#### 3.2.2. The Typical Vacuum Infusion Process Consists of the Following Steps

1. Preparation of the fiber reinforcement: cleaning and orienting the natural fibers to optimize flow paths.
2. Resin infusion: drawing resin into the preform under vacuum, enabling uniform saturation of the fiber bed.
3. Curing: allowing the impregnated composite to harden, typically at room or elevated temperature.
4. Demolding and post-curing: Removing the composite from the mold and, if required, further conditioning.

Vacuum infusion methods such as VARTM, RIFT, and SCRIMP enable the production of lightweight, high-performance biocomposites with improved mechanical properties and reduced processing defects. Their compatibility with natural fiber fabrics and ability to minimize resin waste make them attractive for sustainable industrial applications, including automotive panels, marine structures, and wind turbine blades

### 3.3. Injection Molding for the Production of Fiber-Reinforced Biocomposites

**Figure 6:**
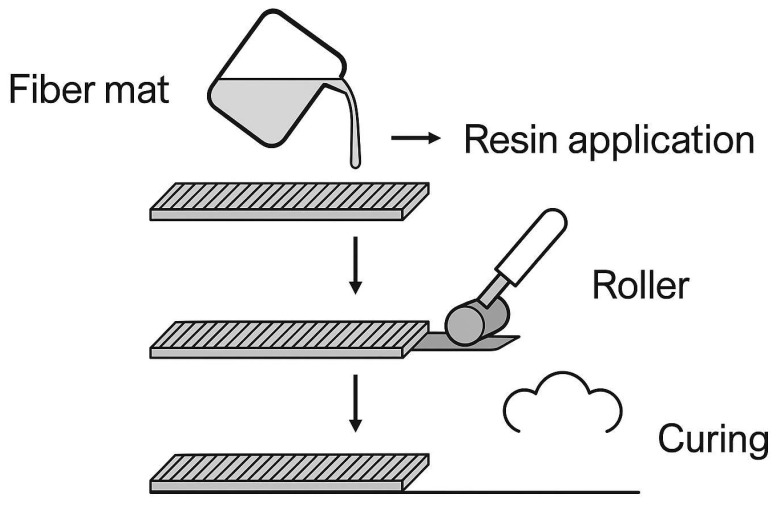
Schematic diagram of the hand lay-up process for fabricating fiber-reinforced composites, showing sequential steps: (1) placement of fiber reinforcement (e.g., natural fiber mats) into the mold, (2) manual application of polymer resin, (3) consolidation using a hand roller to remove air voids and ensure uniform impregnation, and (4) curing under ambient or controlled conditions to solidify the composite structure [76].

Injection molding is one of the most industrially significant techniques for the large-scale fabrication of fiber-reinforced biocomposites, particularly those based on thermoplastic matrices. In this process, polymeric materials, typically in the form of pellets or granules, are introduced into a heated barrel, where they are melted and injected into a mold under high pressure. After injection, the material cools and solidifies within the mold cavity, replicating the geometry with high dimensional precision. Although primarily used with thermoplastics, the method can also be adapted to thermosetting systems through reactive injection techniques. The key components of the injection molding system include the injection unit (for polymer melting and injection), the clamping unit (for mold closure), and the mold assembly itself. The injection unit ensures consistent heating and homogeneous flow of the polymer under controlled pressure, allowing complete filling of the mold cavity and minimizing voids. The efficiency of the injection molding process is influenced by several factors, including the design of the mold, the temperature and pressure conditions, and the rheological properties of the polymer being used. Precise control over processing parameters is critical to ensure fiber integrity, particularly in natural fiber-reinforced systems where excessive shear or temperature may lead to degradation. Injection molding machines are typically available in either horizontal or vertical configurations, each suitable for different production needs. Horizontal configurations are preferred for high-speed mass production, while vertical machines are used for inserting molding or low-volume specialized parts. Proper synchronization of machine components ensures uniform material distribution, reproducibility, and mechanical performance of the final product. Injection molding is particularly well suited for manufacturing high-precision composite components with complex geometries, tight tolerances, and high throughput demands. This makes it a preferred method in the automotive, electronics, and packaging industries, where biocomposites are increasingly replacing traditional materials. To enhance the understanding of the manual lamination approach discussed above, Figure 6 presents a schematic diagram of the hand lay-up process, which remains one of the most accessible and widely used techniques for producing fiber-reinforced composites. The diagram illustrates the typical sequence of operations: initial placement of natural fiber reinforcements into an open mold, manual application of the polymer resin to impregnate the fibers, consolidation using a hand roller to eliminate air entrapment and achieve uniform wetting, and finally, ambient or thermally assisted curing of the composite structure. This method is particularly advantageous for fabricating large, flat, or moderately contoured components without the need for expensive equipment. Figure 6 serves as a visual complement to the text, clarifying operational steps and emphasizing the importance of proper manual control to ensure repeatability and structural integrity in composite production [76].

### 3.4. Pultrusion Process for Fiber-Reinforced Composite Production

The pultrusion process is a highly effective continuous manufacturing technique utilized for producing fiber-reinforced composite materials. These materials are characterized by superior mechanical properties and uniform fiber distribution. In this process, continuous fiber reinforcements including glass, carbon, and natural fibers are drawn through a resin bath and subsequently pulled through a heated die, where the resin undergoes polymerization. This results in the formation of a rigid, durable composite material with optimized performance characteristics [77]. Unlike discontinuous molding methods, pultrusion ensures a constant fiber volume fraction and alignment, which directly translates to enhanced longitudinal strength and predictable mechanical behavior.

#### 3.4.1. Characteristics of the Pultrusion Process:

- Continuous Manufacturing:
  Pultrusion is a continuous process, making it highly suitable for the mass production of composite materials, particularly those with long dimensions, such as pipes and beams, which require consistent fiber reinforcement throughout the structure [78].
- Uniform Fiber Distribution:
  The continuous fibers are uniformly distributed within the composite matrix, ensuring that the material exhibits optimal mechanical properties, particularly in the longitudinal direction. This makes pultruded biocomposites particularly advantageous in load-bearing applications, where high stiffness-to-weight ratios are required [79].
- High-Performance Composites:
  The pultrusion process results in composites with excellent dimensional accuracy, high resistance to environmental degradation, and superior mechanical properties. Additionally, the closed-die design minimizes emissions and improves worker safety, making the process suitable for environmentally conscious production of sustainable materials.

#### 3.4.2. Process Parameters:

- Temperature Control:
  The resin within the heated die is subjected to temperatures ranging between 150 °C and 200 °C, depending on the resin type and the specific end-use requirements. These elevated temperatures must be precisely controlled, especially when using thermally sensitive natural fibers, to avoid degradation and preserve fiber integrity [80].
- Pressure Application:
  The pultrusion process operates under controlled pressure conditions, typically ranging from 2 to 10 MPa. This ensures effective mold filling and optimal consolidation of the resin and fiber matrix [81].
- Pulling Speed:
  The speed at which the fibers are drawn through the resin bath and die is adjusted to control the material’s properties. The pulling speed generally varies from 0.5 m/min to 2 m/min, depending on the desired fiber volume fraction and the composite’s final thickness [82,83,84,85].

The pultrusion process has found widespread application in the fabrication of structural composite components, encompassing pipes, beams, reinforced construction profiles, and bridge components. In the context of plant-fiber-reinforced composites, pultrusion enables consistent integration of lignocellulosic reinforcements (e.g., flax, hemp, jute) into profiles requiring high tensile strength, especially when appropriate surface treatments are used to improve resin–fiber adhesion. Moreover, pultrusion plays a critical role in various industries, such as aerospace, automotive, and construction, where the use of composite materials is imperative for their ability to exhibit superior mechanical strength, high corrosion resistance, and low weight. Despite its advantages, the pultrusion of bio-based fibers may face challenges related to resin compatibility, fiber swelling, and moisture content control, which must be addressed through optimized preprocessing (e.g., drying) and sizing agents. The precision, efficiency, and cost-effectiveness of pultrusion position it as a prominent method for the large-scale manufacturing of high-performance composite materials, offering a sustainable alternative to traditional materials such as steel and concrete, particularly in applications where high strength-to-weight ratios are essential [86].

### 3.5. Compression Molding of Plant-Fiber-Reinforced Polymer Composites

**Figure 7:**
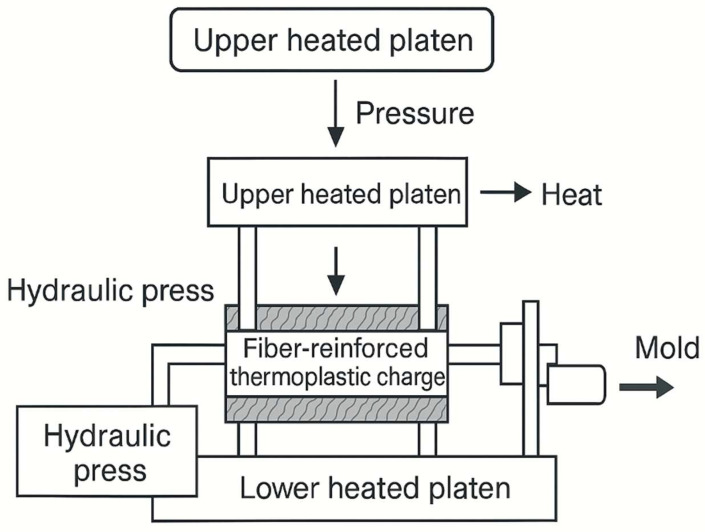
Schematic diagram of the compression molding process for thermoplastic biocomposites. The process involves the placement of a preheated fiber–polymer charge between heated mold platens, followed by the application of pressure and heat to ensure proper flow, impregnation, and consolidation. After cooling, the solidified composite part is removed from the mold [88].

Compression molding is a widely adopted method for producing fiber-reinforced thermoplastic composites, especially when manufacturing medium-to-large components requiring precise geometry, low porosity, and high strength-to-weight ratios. The process involves placing a preheated charge of fiber–polymer material into an open, heated mold cavity. Once the mold is closed, uniform pressure is applied to allow the molten polymer to flow and impregnate the fibers throughout the cavity. This process is relatively straightforward and discontinuous, involving the placement of the mold between the metal plates of a hydraulic press (Figure 7) [11]. For thermoplastics, the material must be sufficiently heated to achieve melt flow, followed by pressure application to ensure fiber wetting and cavity filling. A subsequent cooling step is necessary to solidify the part before demolding. By contrast, thermoset matrices cure chemically under heat and pressure without requiring cooling, forming permanent crosslinks during the pressing cycle. Compression molding is compatible with a wide range of biopolymer matrices (e.g., PLA, PHB) and natural fibers (e.g., flax, jute, kenaf), especially when using mats, preforms, or granulated compounds. The compression molding process is contingent upon the meticulous regulation of several critical parameters, including composite density, thickness, dimensional accuracy, heating and cooling times, temperature, and pressure. Typical processing conditions involve mold temperatures of 160–220 °C, pressures ranging from 2 to 15 MPa, and cycle times adjusted to matrix viscosity and part thickness. Maintaining optimal temperature and pressure profiles is essential to minimize voids, ensure complete fiber impregnation, and achieve uniform mechanical performance across the molded part [87]. To complement the description of the compression molding technique, a schematic representation of the process is presented in Figure 7. The diagram illustrates the sequential stages involved, beginning with the placement of a preheated fiber–polymer charge between heated mold plates, followed by the application of uniform pressure to ensure adequate material flow and fiber impregnation. Subsequent cooling solidifies the molded part before demolding. This visual representation provides a step-by-step overview of the compression molding cycle and enhances understanding of the key operational parameters, namely, temperature, pressure, and cycle time, which directly influence the quality and reproducibility of biocomposite components. The figure serves as a didactic tool to reinforce the relevance of this method in the industrial production of plant-fiber-reinforced thermoplastic composites [88].

### 3.6. Thermal Stability of Reinforcing Fibers

**Figure 8:**
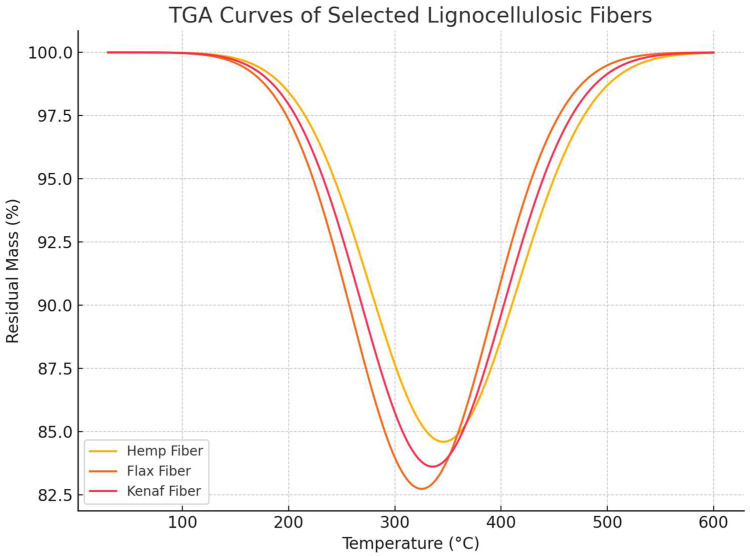
Thermogravimetric analysis (TGA) curves of selected lignocellulosic fibers: hemp, flax, and kenaf. The curves illustrate the thermal degradation behavior under nitrogen atmosphere, showing initial thermal stability up to ~250 °C, major mass loss associated with hemicellulose and cellulose decomposition, and residual char formation above 500 °C.

To investigate the thermal resistance of the reinforcing agents prior to composite processing, thermogravimetric analysis (TGA) was performed for three representative lignocellulosic fibers: hemp, flax, and kenaf. As presented in Figure 8, all tested fibers displayed a characteristic two-step degradation pattern. The initial mass loss below 100 °C corresponds to the desorption of physically bound water. The main degradation phase, occurring between 250 °C and 400 °C, reflects the thermal decomposition of hemicellulose and cellulose components. Hemp fibers exhibited the highest onset temperature for major weight loss, suggesting greater thermal resistance compared to flax and kenaf. Residual char content above 500 °C was notably higher in hemp, which may be linked to its slightly elevated lignin fraction. These findings confirm the feasibility of processing these fibers with thermoplastic matrices such as PLA under conventional extrusion and molding conditions, provided that processing temperatures do not exceed ~230 °C.

## 4. Structure of Cellulose in Plant-Fiber-Reinforced Composites

In order to gain a comprehensive understanding of plant-fiber-reinforced composites, it is crucial to first examine the basic polymeric structure of the natural fibers used in these composites. Most plant-derived fibers are primarily composed of cellulose, hemicellulose, and lignin, with cellulose acting as the principal load-bearing component responsible for their structural integrity. The primary structural component of plant fibers is cellulose, a biopolymer that plays a fundamental role in providing strength and stability to the fiber [89]. Cellulose is a linear homopolysaccharide composed of β-D-glucopyranose units connected via β-(1→4)-glycosidic bonds. The polymer chains organize into microfibrils that exhibit both crystalline and amorphous domains. The crystalline domains, formed by extensive intra- and inter-molecular hydrogen bonding, confer high tensile strength, stiffness, and resistance to enzymatic and chemical degradation. In contrast, amorphous regions possess disordered chain alignment and exhibit greater chemical accessibility and flexibility [90]. The alternation in molecular orientation between glucose residues contributes to the linearity and close packing of chains, which reinforces microfibril rigidity. The amorphous regions contain free hydroxyl (-OH) groups that impart hydrophilicity and facilitate moisture absorption, which can compromise interfacial compatibility with hydrophobic polymers. This challenge makes it necessary to modify the fibers through surface treatments to improve the fiber–matrix adhesion in the final composite [91].

Comparison of Crystalline and Amorphous Domains

The supramolecular architecture of cellulose microfibrils can be divided into two primary regions: crystalline and amorphous. The crystalline regions consist of tightly packed and aligned cellulose chains that are less permeable and highly resistant to hydrolysis, while the amorphous regions are disordered, hydrophilic, and more susceptible to chemical modification [92]. In the amorphous regions, the hydroxyl groups are more exposed, facilitating the absorption of water and other chemicals. The ratio between these two domains determines the fiber’s mechanical performance, swelling behavior, and degradation kinetics in composite environments.

Challenges with Hydrophilicity

The hydrophilic nature of the amorphous regions in cellulose can influence the water retention properties of plant fibers in composites, potentially impacting their performance. Moisture uptake can induce fiber swelling, delamination at the fiber–matrix interface, and a consequent decrease in mechanical integrity. When exposed to moisture, cellulose has been observed to absorb water, which can result in swelling and a reduction in the mechanical properties of the composite.

This phenomenon presents a significant challenge when utilizing plant fibers within hydrophobic polymer matrices. To address this challenge, surface modifications are employed, including chemical treatments and the application of coupling agents. These modifications are intended to reduce the fiber’s affinity for water and enhance the bonding between the fiber and the matrix [93].

Impact on the Fiber–Matrix Interface

The crystalline regions of cellulose, being more resistant to chemical attack, can be difficult to penetrate by resins or polymer matrices. This limited accessibility hinders resin infiltration and interdiffusion, which are critical for strong interfacial adhesion. This makes it harder for the fibers to form strong bonds with the matrix, potentially limiting the composite’s overall performance. Therefore, improving the fiber–matrix interface is a critical aspect of designing efficient plant-fiber-reinforced composites. Strategies like mercerization, acetylation, and other chemical treatments are often employed to enhance fiber–matrix compatibility by modifying the surface of the fibers, reducing their hydrophilicity, and improving their adhesion to the matrix [94]. A comprehensive understanding of the structural and compositional characteristics of cellulose and other components present in plant fibers is imperative for the effective design of plant-fiber-reinforced composites. Targeted chemical and physical modifications of cellulose-rich fibers enable improved performance in composite systems, particularly through enhanced interfacial bonding and moisture resistance. Modifying the fiber structure and enhancing the fiber–matrix interface through chemical treatments has been shown to significantly improve the mechanical properties and thermal stability of the composites. As the demand for sustainable materials increases, plant fibers offer an attractive alternative to traditional synthetic fibers, provided that their structural and chemical characteristics are carefully controlled [95].

### 4.1. Chemical Modifications of Plant Fibers

Chemical modifications of plant fibers are essential for improving their properties by tailoring surface characteristics, polarity, and interfacial behavior, particularly for optimizing composite performance. Natural plant fibers, which are predominantly composed of cellulose, inherently possess hydrophilic properties due to the presence of hydroxyl (-OH) groups within their structural composition. Consequently, these fibers frequently exhibit suboptimal adhesion when bonded to hydrophobic polymer matrices. Surface chemical treatments are therefore applied to reduce water uptake, increase surface roughness, and introduce reactive functional groups that enhance wettability and chemical bonding with the polymer matrix. Through the implementation of diverse chemical treatments, the mechanical, thermal, and environmental properties of plant fibers can be tailored to enhance their functionality [96]. The most prevalent chemical modifications encompass scouring, bleaching, mercerization, acetylation, benzoylation, and the incorporation of coupling agents. Each of these processes plays a distinct role in enhancing the fiber’s functionality, making it more suitable for composite applications. Scouring and bleaching are initial purification steps aimed at removing non-cellulosic impurities, such as waxes, pectins, and surface-bound oils. Scouring involves the treatment of fibers with alkaline solutions, such as sodium hydroxide (NaOH), to remove contaminants including oils, pectins, and waxes. Subsequent to the scouring process, the utilization of oxidizing agents, such as hydrogen peroxide (H_2_O_2_) or chlorine-based compounds, is often employed to eliminate residual coloration and surface impurities. These processes not only enhance the surface appearance of the fibers but also increase their purity, rendering them more receptive to bonding with polymer matrices. Mercerization, a treatment in which plant fibers are immersed in a concentrated sodium hydroxide (NaOH) solution, modifies the cellulose structure, increasing its crystallinity and improving its ability to bond with polymers [97]. This process leads to fiber swelling, which increases the surface area and enhances the fibers’ compatibility with hydrophobic matrices. Mercerization also disrupts hydrogen bonding in the crystalline regions, partially converting cellulose I to cellulose II, which results in improved dimensional stability and greater flexibility in interfacial interaction. Beyond enhancing adhesion, mercerization has been shown to augment the fiber’s mechanical strength, dimensional stability, and resistance to shrinkage. Consequently, mercerized fibers are particularly advantageous in high-performance composite applications, where durability and resistance to deformation are paramount. Acetylation, a significant chemical modification, involves the introduction of acetyl groups (-COCH_3_) into the cellulose structure.

This process reduces the fiber’s hydrophilicity by modifying the hydroxyl groups, making the fibers more resistant to moisture absorption and improving their resistance to chemical degradation. Acetylated fibers demonstrate enhanced durability, particularly in environments characterized by high humidity or exposure to chemicals, and exhibit augmented thermal stability [98]. Acetylation lowers the polarity of the fiber surface and suppresses hydrogen bonding, making fibers more compatible with apolar or weakly polar thermoplastic matrices such as polyethylene and polypropylene. Benzoylation, a process that introduces benzoyl groups into the cellulose structure, has been shown to enhance the fiber’s hydrophobicity and resistance to environmental factors such as moisture and ultraviolet (UV) radiation.

This process enhances the durability and stability of the fibers, rendering them more suitable for outdoor and industrial applications where exposure to the elements is a concern. Coupling agents, such as silanes or other functional chemicals, are frequently employed to enhance the fiber–matrix interaction by establishing chemical bonds between the fiber and polymer [99]. Silane coupling agents, for example, can form covalent bridges between hydroxyl groups on the fiber surface and reactive groups on the matrix polymer, improving stress transfer at the interface and reducing interfacial failure under load. These agents enhance the mechanical properties of the composite by providing a stronger bond between the fiber and the matrix, thus improving the overall performance of the biocomposite. Furthermore, chemical modifications have been shown to play a crucial role in improving the thermal stability of plant fibers. Lignocellulosic fibers, including those derived from flax, hemp, and jute, typically begin to degrade at temperatures approximating 240 °C. However, the degradation temperatures of individual components, such as cellulose, lignin, and hemicellulose, exhibit significant variation. Lignin demonstrates the greatest sensitivity to heat, with a degradation temperature of approximately 200 °C. In contrast, cellulose and hemicellulose begin to degrade at higher temperatures [100]. Chemical processes such as acetylation and lignin extraction can increase thermal resistance by eliminating low-stability components or introducing thermally stable substituents, thereby enabling processing at elevated temperatures during extrusion, compression, or injection molding. Such modifications also increase matrix compatibility and broaden the range of potential applications in demanding industrial environments as summarized in Table 4.

Chemical modifications of plant fibers are imperative for customizing their properties to satisfy the particular requirements of composite materials. These modifications enhance the compatibility of the fibers with polymer matrices, augment their mechanical and thermal properties, and increase their durability and resistance to environmental factors. Consequently, chemically modified plant fibers are well-suited for use in biocomposites that demand high performance, sustainability, and durability across a broad spectrum of applications. Further optimization of modification techniques will enable the development of next-generation bio-based composites with superior interfacial integrity, reduced water sensitivity, and improved processability. The refinement of these modification processes has the potential to expand the application of plant-fiber-based composites, contributing to a more sustainable and efficient future for material science [103].

### 4.2. Chemical Interactions Between Plant Fibers and Polymer Matrices

Chemical modifications of plant fibers are essential processes to enhance the properties of these fibers by improving their interfacial compatibility with polymer matrices through surface polarity reduction and functionalization. The intrinsic polarity mismatch between hydrophilic lignocellulosic fibers and typically hydrophobic thermoplastic matrices often leads to poor interfacial adhesion and inefficient stress transfer in biocomposites. Consequently, chemical surface modifications are implemented to introduce functional groups, improve wettability, and facilitate covalent or hydrogen bonding across the fiber–matrix interface. These modifications aim to improve mechanical properties, thermal stability, and environmental resistance, while also enhancing the interfacial bonding between the fiber and the polymer matrix. The most common chemical modifications applied to plant fibers include alkali treatment, silane coupling, acetylation, peroxide treatment, and bleaching [104,105,106]. Each of these methods modifies the surface characteristics of the fibers, facilitating stronger fiber–matrix bonding and enhancing the overall performance of the composite. Below, the underlying mechanisms and benefits of each treatment are discussed in detail.

Alkali Treatment of Plant Fibers

Alkali treatment is a prevalent method for modifying plant fibers, particularly cellulose fibers. The process involves the immersion of the fibers in an aqueous sodium hydroxide (NaOH) solution, which disrupts hydrogen bonds in the cellulose network and removes amorphous, non-cellulosic materials such as hemicellulose, pectin, and lignin. By partially removing matrix-inhibiting components and increasing surface roughness and hydroxyl group availability, the fiber becomes more reactive toward matrix polymers.

Furthermore, alkali treatment introduces reactive hydroxyl groups (-OH), which enhance the fiber’s bonding capabilities with the matrix [107,108].

Reaction:
  Cell–OH + NaOH → Cell–O^−^Na^+^ + H_2_O + extracted impurities

Although crystallinity may decrease, improved fiber accessibility enhances interfacial reactivity and wetting behavior in composite systems.

#### 4.2.1. Acetylation of Plant Fibers

Acetylation involves introducing acetyl groups (–COCH_3_) to the hydroxyl sites of cellulose using acetic anhydride or acetyl chloride. This substitution lowers fiber polarity, thereby reducing water absorption and enhancing compatibility with hydrophobic matrices such as PP, PE, or PLA. The esterification reaction modifies hydrogen-bonding networks and limits fiber swelling during moisture uptake, thereby increasing dimensional stability.

Reaction:
  Cell–OH + (CH_3_CO)_2_O → Cell–O–COCH_3_ + CH_3_COOH

Acetylated fibers demonstrate enhanced durability, particularly in moisture-prone environments or chemically aggressive applications [109].

#### 4.2.2. Peroxide Treatment

Peroxide treatment utilizes organic peroxides such as benzoyl peroxide or dicumyl peroxide to generate free radicals that initiate crosslinking between the fiber and matrix. Radicals abstract hydrogen atoms from hydroxylated surfaces or polymer chains, promoting the formation of covalent bridges that reinforce interfacial adhesion.

Initiation Mechanism:
  RO–OR → 2RO•; RO• + CH– (Polymer) → crosslinked structure

Peroxide treatment enhances the reactivity and bonding of the fiber, thereby improving the tensile and impact strength of the resulting composite [110,111,112].

#### 4.2.3. Bleaching with Sodium Chlorite

Bleaching is employed to remove residual lignin, pigments, and extractives that hinder fiber–matrix interaction. Sodium chlorite (NaClO_2_) in acidic medium generates chlorine dioxide (ClO_2_), which oxidizes lignin and associated non-cellulosic residues. The process not only increases surface cleanliness but also exposes more cellulose domains available for bonding, improving fiber uniformity and color.

Reaction:
  Lignin + ClO_2_ → oxidized lignin fragments + water-soluble by-products

By removing these impurities, the fiber becomes more compatible with the polymer matrix, improving its overall performance in composite applications [113]. The chemical modifications of plant fibers are imperative for enhancing their compatibility with polymer matrices, improving their mechanical properties, and ensuring better durability and stability in biocomposites. Through methods such as alkali treatment, silane coupling, acetylation, peroxide treatment, and bleaching, plant fibers can be optimized for composite applications. These treatments alter surface energy, remove barriers to adhesion, and create favorable conditions for mechanical interlocking and chemical bonding at the interface. As a result, plant-fiber-reinforced materials are a promising and sustainable alternative to synthetic fibers in various industrial sectors. Ongoing advances in fiber functionalization, especially in environmentally benign treatment chemistries, will further expand the range and reliability of bio-based composites in structural and semi-structural applications [114].

### 4.3. Mechanical Properties of Plant-Fiber-Reinforced Composites

The mechanical properties of plant fibers are critical in determining the performance of plant-fiber-reinforced composites (BFCs). These properties are influenced by several factors, including fiber length, alignment, aspect ratio, distribution within the matrix, and the strength of the fiber–matrix interface [115]. Under external loading, the fibers act as the primary load-bearing elements, and the stress transfer from matrix to fiber is governed by the degree of interfacial adhesion and the orientation of the reinforcement relative to the direction of force. The load-bearing capacity of the fibers is predominantly influenced by their aspect ratio, the direction of load application, and molecular orientation.

Additionally, the effectiveness of fiber dispersion and wetting by the matrix significantly influences the mechanical integrity of the composite. In composites with longitudinal fiber orientation, the fibers are aligned along the principal loading axis. This configuration results in high tensile strength because the fibers are aligned in the direction of the applied load, allowing for efficient axial stress transfer. However, the compressive strength is often lower due to the tendency of slender fibers to buckle under axial compression. In contrast, diagonal fiber arrangements provide improved resistance to multi-axial loading conditions. While the axial tensile strength is lower than in purely longitudinal systems, diagonal orientation enhances the composite’s performance under off-axis or combined loading scenarios. Randomly oriented fibers confer isotropic mechanical behavior in the plane of the composite and improve energy absorption during impact or vibration. Although the tensile strength in a specific direction may be limited, this configuration is advantageous in damping applications or in complex geometries where directionally aligned reinforcement is impractical [116]. Thus, fiber orientation is a critical design parameter that must be tailored to the intended mechanical performance of the composite system. The fiber length is another key factor in determining mechanical performance.

Longer fibers offer a higher surface area for stress transfer and better reinforcement efficiency. Critical fiber length (l_p_) must be exceeded to ensure effective load transfer; otherwise, fibers act as stress concentrators and compromise composite strength. In contrast, short fibers, while easier to process, contribute less to tensile and flexural strength due to limited bonding surface and higher fiber pull-out probability. Mechanical reinforcement also depends on the load application direction. Composites loaded parallel to the fiber axis demonstrate significantly higher tensile modulus and failure stress compared to those loaded transversely [109]. The last and arguably most important factor is the quality of the fiber–matrix interface. A strong interface ensures efficient stress transfer and suppresses debonding, fiber pull-out, and crack propagation. A well-bonded interface facilitates stress transfer and improves the durability of the material, while a weak interface can lead to premature failure. Interfacial adhesion can be enhanced through physical roughening, chemical treatments (e.g., silanization or acetylation), or compatibilizer incorporation. These treatments reduce interfacial tension and improve wetting by the matrix polymer. Such modifications are important for ensuring that the composite performs well under load and maintains its structural integrity over time. Therefore, a holistic optimization of fiber architecture, surface functionality, and processing technique is required to develop biocomposites with competitive and tunable mechanical properties suitable for engineering applications [110,111,112], as summarized in Table 5.

## 5. Factors Influencing the Performance and Longevity of Plant-Fiber-Reinforced Biocomposites

Biocomposites, which integrate natural plant fibers as reinforcement in polymer matrices, offer significant advantages such as biodegradability, renewability, and application versatility. However, the long-term performance of these materials is constrained by environmental sensitivity, particularly to moisture and biological degradation. The intrinsic hydrophilicity of plant fibers, primarily due to the abundance of hydroxyl (-OH) groups in cellulose, hemicellulose, and lignin, leads to moisture absorption and subsequent degradation of interfacial bonding in the composite structure [117]. Upon exposure to humid environments, water molecules penetrate the fiber structure and accumulate at the fiber–matrix interface, leading to fiber swelling, microcrack formation, and debonding from the matrix. This disruption of the fiber–matrix bond leads to a decline in tensile strength, compressive strength, and dimensional stability of the composite. These changes are often irreversible and are especially detrimental in load-bearing or outdoor applications subjected to cyclic humidity and thermal conditions. The susceptibility of plant fibers to biological degradation is another key factor that limits the longevity of biocomposites. Microorganisms such as bacteria and fungi can degrade cellulose by enzymatic hydrolysis, compromising the structural integrity of the fiber and accelerating mechanical failure. Moisture not only acts as a plasticizer but also promotes microbial colonization, further intensifying degradation. Therefore, enhancing biocomposite durability requires a multipronged approach, involving both chemical fiber treatments and matrix-level modifications. One highly effective strategy is the chemical modification of the fiber surface. Treatments such as alkali pretreatment, acetylation, and silane grafting reduce fiber polarity, eliminate amorphous regions, and introduce functional groups for better interaction with hydrophobic polymers [118]:

- Alkali treatment disrupts hydrogen bonding in the cellulose microfibrils and removes hemicellulose and lignin, exposing reactive –OH groups.
- Acetylation substitutes hydroxyl groups with acetyl moieties, which limits hydrogen bonding with water.
- Silane coupling agents form covalent bridges between fiber and matrix phases, drastically improving interfacial adhesion.

These treatments not only improve dimensional stability under humidity but also suppress fiber pull-out and delamination under load. In addition to fiber modification, the choice of matrix polymer is critical. Hydrophobic matrices such as polyethylene (PE), polypropylene (PP), or vinyl ester resin exhibit reduced moisture uptake, while thermosetting systems can offer superior dimensional stability and chemical resistance when properly cured. Adding stabilizers, antioxidants, or antimicrobial agents to the polymer matrix further extends durability by suppressing microbial growth and oxidative degradation [119].

**Figure 9:**
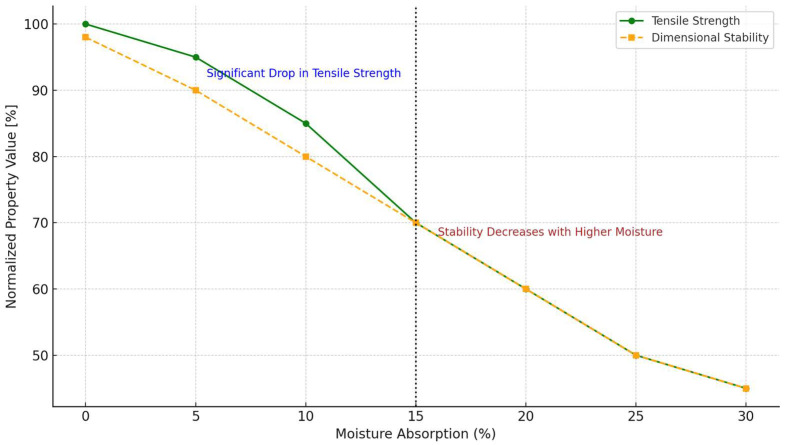
Effect of moisture absorption on the normalized tensile strength and dimensional stability of plant-fiber-reinforced composites. Both properties exhibit a progressive decline with increasing moisture content, with tensile strength showing a particularly sharp decrease beyond 10–15% absorption. The vertical dotted line marks the threshold above which structural performance degradation becomes critical.

The effect of moisture absorption on the mechanical and dimensional behavior of plant-fiber-reinforced composites is illustrated in Figure 9. As shown, both tensile strength and dimensional stability decrease as moisture uptake increases. Notably, tensile strength experiences a rapid decline beyond 10–15% moisture content, highlighting the sensitivity of fiber–matrix adhesion and microstructural integrity to water ingress [120]. Dimensional changes follow a more gradual trend but become significant above the critical threshold, suggesting matrix swelling and fiber plasticization effects. These findings underscore the importance of moisture resistance strategies in composite design, particularly for applications in humid environments [121,122].

## 6. Sustainable Applications of Plant-Fiber-Reinforced Composites: Advances and Prospects

### 6.1. Thermoplastic Processing

Plant-fiber-reinforced composites (PFRP), primarily derived from cellulose-based fibers, are increasingly becoming a cornerstone in the development of sustainable materials. These composites utilize natural fibers like hemp, flax, jute, and sisal, offering excellent mechanical properties, low density, and minimal environmental impact. Among their ecological advantages are biodegradability, reduced energy consumption during production, and lower carbon footprints, positioning them as promising replacements for synthetic composites in a variety of industrial applications, including automotive, construction, packaging, and biomedical sectors [75]. At the molecular level, cellulose—a linear polysaccharide composed of β(1→4) D-glucose units—acts as the structural backbone of plant fibers and is responsible for their high tensile strength, rigidity, and renewability [123]. This structure provides plant fibers with high tensile strength and rigidity, making them suitable as reinforcements in composite materials. Cellulose’s crystalline nature contributes to its strong mechanical properties, while its hydrophilic characteristics—due to the presence of hydroxyl groups—can make plant fibers prone to moisture absorption, which may affect the dimensional stability and overall performance of the composites in humid environments. Consequently, moisture absorption may lead to fiber swelling, weakening the fiber–matrix interface and reducing mechanical performance over time. However, various chemical treatments, such as mercerization, acetylation, and the use of silane coupling agents, are employed to modify the fibers’ surface properties, improving their compatibility with polymer matrices and enhancing their mechanical and thermal performance [124]. In addition to cellulose, hemicellulose and lignin—two other main constituents—play vital roles in defining the fiber’s behavior. Hemicellulose, a branched polysaccharide, contributes to the flexibility of the fiber, while lignin, an aromatic polymer, binds the fibers together and provides rigidity and resistance to microbial degradation. However, excessive lignin content can impair fiber–matrix interaction, so partial removal is often necessary to improve interfacial bonding and composite durability. The development of plant fiber composites has led to innovations across various sectors. The automotive industry, for instance, is utilizing these biocomposites for lightweight components that reduce vehicle weight, enhance fuel efficiency, and lower CO_2_ emissions. Companies like General Motors, BMW, and Toyota have integrated plant-fiber-based materials in various car parts, such as seat backs, headliners, and door panels [125]. Their low density combined with sufficient impact resistance makes them ideal candidates for semi-structural applications. The potential of these materials is also evident in the construction sector, where biocomposites are used for thermal insulation, offering energy-efficient solutions in building envelopes. They exhibit favorable acoustic insulation properties and fire retardancy when properly modified. The biomedical field has also seen advances, with biocomposites being used for applications like wound dressings, tissue scaffolds, and biosensors due to their biocompatibility and biodegradability. Notably, recent progress in surface functionalization and sterilization compatibility has made PFRPs attractive for controlled drug delivery and temporary implants. Sustainability remains the driving force behind the expanding use of biocomposites. Compared to synthetic materials like glass fibers and petroleum-derived plastics, biocomposites made from plant fibers require less energy to manufacture and release fewer greenhouse gases throughout their lifecycle. The Life Cycle Assessment (LCA) approach is used to evaluate the environmental impact of biocomposites at each stage of their life cycle, ensuring that they meet sustainability criteria. LCA studies consistently demonstrate the reduced ecological footprint of plant fiber composites, particularly when locally sourced raw materials and recyclable polymers are employed.

### 6.2. Thermoset Processing

The increasing demand for eco-friendly products is pushing industries toward the use of renewable materials, and the role of plant fibers in this shift is undeniable. The global market for biocomposites is expected to continue growing, driven by consumer preferences for sustainable products and the need to comply with environmental regulations. This growth is further supported by innovations in composite production techniques, making biocomposites more cost-effective and accessible for widespread use. For example, developments in extrusion compounding and additive manufacturing (3D printing) are enabling the efficient fabrication of customized plant-based composites with tailored properties. The combination of natural and synthetic materials in biocomposites allows for the production of high-performance, low-cost solutions that meet the evolving needs of various industries. The future of plant fiber composites will likely involve greater integration of green chemistry principles, including the use of bio-based matrices and minimal use of toxic chemicals in fiber treatments. As research into the optimization of plant-fiber properties and their compatibility with different matrices continues, the range of applications for biocomposites will expand [85,126,127,128,129]. Their ability to offer lightweight, durable, and environmentally friendly solutions makes them a key component in the shift towards a more sustainable, eco-conscious future. By minimizing environmental burdens without compromising structural or mechanical performance, PFRPs are positioned as essential materials in sustainable design and engineering, as summarized in Table 6.

## 7. Conclusions

In response to the mounting demand for sustainable materials, plant-fiber-reinforced composites (PFRP) have garnered considerable attention in a variety of industrial applications. These composites, which are manufactured from natural fibers such as hemp, flax, jute, and sisal, offer a number of ecological and economic advantages, including biodegradability, recyclability, low production costs, and high mechanical strength. The primary structural component of these fibers, cellulose, provides outstanding tensile strength and rigidity, while hemicellulose and lignin contribute to their flexibility and stability. Plant fiber composites have found widespread application in a variety of industries, including the automotive, construction, and biomedical sectors. Within the automotive sector, these composites find application in lightweight components such as door panels, seat backs, and headliners. The incorporation of plant-based materials in this manner contributes to a reduction in vehicle weight, enhancement of fuel efficiency, and reduction of CO_2_ emissions. In the field of construction, biocomposites are utilized in the fabrication of thermally insulating materials for energy-efficient buildings. In the biomedical field, plant-based composites find application in wound dressings, tissue scaffolds, and biosensors due to their biocompatibility and biodegradability. The structural properties of plant fibers, including cellulose content, molecular orientation, and interactions with polymer matrices, have a significant impact on the mechanical performance of biocomposites. Nevertheless, their long-term performance is hindered by inherent hydrophilicity and susceptibility to microbial degradation, which affect dimensional stability and mechanical integrity. To address these challenges, various chemical treatments are applied, including mercerization, acetylation, and the use of coupling agents (e.g., silanes). These treatments are designed to improve fiber–matrix compatibility, enhance durability, and prevent degradation over time. It is noteworthy that plant fibers exhibit hydrophilic properties due to the presence of hydroxyl groups in their molecular structure. This hydrophilicity renders them susceptible to moisture absorption, which can result in dimensional instability and diminished interfacial adhesion between fibers and polymer matrices. Consequently, surface modifications, such as alkali treatment, are frequently employed to enhance the moisture resistance of fibers and improve their overall performance in composites. Moreover, advances in processing techniques—such as injection molding, vacuum infusion, and compression molding—enable scalable production of high-performance biocomposites with consistent quality. The demand for sustainable materials is a driving force for the adoption of biocomposites across various sectors. Biocomposites derived from plant fibers exhibit a reduced environmental impact in comparison to synthetic materials, as evidenced by a diminished carbon footprint and a reduced energy consumption during the production process. Life Cycle Assessments (LCAs) of biocomposites demonstrate their environmental benefits, from raw material extraction to product disposal, supporting their widespread use in industrial applications. In summary, plant-fiber-reinforced composites represent a viable and scalable approach toward sustainable materials engineering, combining ecological benefits with favorable mechanical performance. To further expand the use of biocomposites and advance their role in a sustainable and eco-friendly future in material science and engineering, continued research is essential. This research should focus on optimizing fiber properties and improving polymer compatibility. Additionally, future work should emphasize cost-effective processing, advanced surface engineering, and durability enhancement under long-term service conditions.

## Figures and Tables

**Table 1:** Representative mechanical properties, density, and cellulose content of selected plant fibers used in polymer composite applications [26,27,28].

Plant Fiber	Tensile Strength (MPa)	Elastic Modulus (GPa)	Density (g/cm^3^)	Cellulose Content (%)
Hemp	550–900	30–70	1.48	70–74
Flax	500–1500	50–70	1.50	65–85
Jute	400–800	10–30	1.46	61–71
Kenaf	400–600	22–60	1.45	60–72
Coir	100–200	4–6	1.20	32–43

**Table 2:** Comparative properties of thermosetting and thermoplastic polymers for biocomposite applications [55].

Property	Thermosetting Polymers	Thermoplastic Polymers
Fiber Interaction	Excellent	Temperature-dependent
Resin Viscosity	Low	High
Recyclability	Challenging	Easily recyclable
Thermal Resistance	High	Moderate

**Table 3:** Comparison of processing techniques for plant-fiber-reinforced polymer composites, including technical advantages, limitations, and application fields [60,61,62,63,64,65,66,67].

Processing Method	Advantages	Disadvantages	Applications
Injection Molding	High throughput, precise shaping, suitable for complex geometries	Requires high pressure and high temperatures, limited to short fibers	Packaging, consumer goods, small technical parts
Vacuum-Assisted Resin Transfer Molding (VARTM)	Reduced voids, accurate fiber control, scalable for large parts	High equipment cost, resin viscosity dependent	Aerospace, automotive, structural composites
Hand Lay-up	Low cost, simplicity, suitable for prototyping	Labor-intensive, high variability, risk of defects	Manual or custom components, prototyping
Pultrusion	Continuous process, uniform fiber distribution, structural quality	Limited to linear profiles, expensive dies	Rods, beams, civil and marine infrastructure
Compression Molding	Short cycle times, well-suited for mats and sheets	Less effective for thick sections or irregular shapes	Automotive interiors, paneling, structural parts

**Table 4:** The chemical modification processes for plant fibers [101,102].

Process	Modification Goal	Effect
Scouring	Removal of organic impurities	Increased fiber purity
Bleaching	Removal of surface impurities	Improved surface appearance
Mercerization	Increase bonding ability with polymers	Enhanced fiber–matrix adhesion and strength
Acetylation	Enhance resistance to moisture/chemicals	Improved durability and thermal stability
Benzoylation	Improve resistance to environmental factors	Increased hydrophobicity and UV resistance

**Table 5:** The influence of fiber orientation on the mechanical performance of plant-fiber-reinforced composites [113].

Fiber Orientation Type	Tensile Strength	Compressive Strength	Advantages
Longitudinal Fibers	High	Low	Highest uniaxial tensile strength
Diagonal Fibers	Medium	Medium	Balanced, multidirectional loads
Random Fibers	Low to Medium	Medium	Impact resistance, flexibility

**Table 6:** Applications of plant-fiber-reinforced composites across various industries [130].

Industry	Application	Example
Automotive	Lightweight components for vehicles	Door panels, seat backs, headliners, floor mats
Construction	Energy-efficient building materials	Insulated panels, eco-friendly building envelopes
Biomedical	Biocompatible materials for healthcare	Wound dressings, tissue scaffolds, biosensors

## Data Availability

Data are contained within the article.
